# Alcohol use and cardiometabolic risk in the UK Biobank: A Mendelian randomization study

**DOI:** 10.1371/journal.pone.0255801

**Published:** 2021-08-11

**Authors:** Joanna Lankester, Daniela Zanetti, Erik Ingelsson, Themistocles L. Assimes

**Affiliations:** 1 Department of Medicine (Division of Cardiovascular Medicine), Stanford University School of Medicine, Stanford, CA, United States of America; 2 GlaxoSmithKline, Inc., Brentford, England, United Kingdom; 3 VA Palo Alto Health Care System, Palo Alto, CA, United States of America; Osaka University Graduate School of Medicine, JAPAN

## Abstract

Observational studies suggest alcohol use promotes the development of some adverse cardiometabolic traits but protects against others including outcomes related to coronary artery disease. We used Mendelian randomization (MR) to explore causal relationships between the degree of alcohol consumption and several cardiometabolic traits in the UK Biobank. Using the well-established *ADH1B* Arg47His variant (rs1229984) and up to 24 additional SNPs recently found to be associated with alcohol consumption in an independent dataset as instruments, we conducted two-stage least squares and inverse weighted variance MR analyses, both as one-sample analyses in the UK Biobank and as two-sample analyses in external consortia. In the UK Biobank inverse variance weighted analyses, we found that one additional drink of alcohol per day was positively associated with systolic blood pressure (beta = 2.65 mmHg [1.40, 3.89]), hemorrhagic stroke (OR = 2.25 [1.41, 3.60]), and atrial fibrillation (OR = 1.26 [1.07, 1.48]), which were replicated in multivariable analyses. Alcohol was also associated with all cardiovascular disease and all-cause death. A positive association with myocardial infarction did not replicate in multivariable analysis, with suggestive mediation through blood pressure; similarly, a positive association between alcohol use with type 2 diabetes was mitigated by BMI in multivariable analysis. Findings were generally null in replication with two-sample analyses. Alcohol was not protective for any disease outcome with any analysis method, dataset, or strata. Stratifications by sex and smoking in the UK Biobank revealed higher point estimates of risk for several outcomes for men and mixed results for smoking strata, but no statistically significant heterogeneity. Our results are consistent with an overall harmful and/or null effect of alcohol on cardiometabolic health at all levels of use and suggest that even moderate alcohol use should not be promoted as a part of a healthy diet and lifestyle.

## Introduction

The relationship between alcohol and cardiovascular disease is important to understand given the high prevalence of alcohol consumption [1, 2]. In decades of epidemiological work, alcohol consumption has shown an inverse or J-shape association with multiple traits related to cardiometabolic health including Type 2 diabetes [3, 4], non-fatal and fatal coronary heart disease [5–7], ischemic stroke [8–10], atrial-fibrillation [11], and congestive heart failure [12].

One interpretation of these relationships has been that moderate drinking is beneficial to cardiometabolic health. A problem with this interpretation is that the relationship is inconsistent with that observed for some known risk factors; for example, alcohol has been directly associated with outcomes such as hypertension irrespective of the degree of intake [13]. A long-standing hypothesis to reconcile these observations stipulates that the negative effects on blood pressure are modest and are surpassed by the positive effects on HDL levels [14, 15] that are either directly affected by alcohol and/or a consequence of an improvement in insulin sensitivity [16]. However, this hypothesis has been challenged in the last decade by multiple randomized control trials of HDL-raising drugs, as well as Mendelian randomization studies that have failed to demonstrate the benefits on risk of cardiovascular disease (CVD) of pharmacologically or genetically raised HDL [17–19].

The extent to which observational studies can shed light on the relationship between alcohol and CVD is questionable due to confounding and reverse causality [20]. Alcohol use is related to cultural, socioeconomic, and lifestyle factors which cannot be fully accounted for in observational analyses. Furthermore, several studies have suggested substantial differences in the effects of alcohol on cardiometabolic traits between men and women [3, 4, 11, 12, 16, 21]. Mendelian randomization (MR) facilitates a comparison of groups of subjects that consume more vs. less alcohol that is free of confounding, allowing us to better understand the causal effect of consumption. In this study, we determine the causal relationship between alcohol and cardiovascular risk factors and disease in the UK Biobank by performing an instrumental variable analysis using a genetic variant in a gene (*ADH1B)* that is known to be responsible for the metabolism of alcohol and associated with the amount of alcohol consumed. We attempt to validate findings using summary statistics from external consortial studies for related phenotypes. Within the UK Biobank, we also take advantage of the large numbers to explore strength of associations stratified by sex and smoking status.

## Methods

### Study cohort

The UK Biobank is a prospective study of over 500,000 participants recruited in 2006–2010 [22]. Data collected from the participants included questionnaires, physical measures, sample assays, genotyping, and ongoing longitudinal hospital records. Participants were enrolled at age 40–69. This research has been conducted using the UK Biobank Resource under Application Number 13721. The Research Ethics Committee reference for UK Biobank is 16/NW/0274. The Stanford IRB reviewed the protocol and determined the research did not include human subjects as defined in 45 CFR 46, nor 21 CFR 56.

### Outcomes and quantitative traits

We extracted systolic and diastolic blood pressure, BMI, waist circumference, and body fat percentage from survey data which included physical measurement at the baseline clinic visit. We obtained lipids, blood count variables, and HbA1C from the biomarkers data. We extracted primary and secondary diagnosis disease outcomes from hospital data for myocardial infarction, stroke (hemorrhagic, ischemic, and any stroke), heart failure, atrial fibrillation, and a composite outcome of all cardiovascular events combined, as well as death from each of these disease outcomes according to the relevant ICD codes (Table A in S1 Text). We derived type 2 diabetes status from a combination of diabetes-related questions and self-reported medications (S1 Text).

### Main exposure and covariates

Our main exposure variable of interest was self-reported alcohol use by number of drinks per week or month and type of drink obtained from the survey data. Use of all types of alcohol was aggregated into total grams of alcohol intake per year [23], which was then transformed into equivalent daily glasses of wine (0, >0–1, >1–2, >2–3, and >3) to facilitate interpretability. Covariates also from survey data included sex, age, region of recruitment, socioeconomic status, ethnicity, smoking status, blood pressure medications, cholesterol-lowering medications, insulin and other diabetes drugs, and fasting status (for biomarkers) (S2 Text).

We used two instrumental variables (IV) for our MR analyses of alcohol use. First, we used the *ADH1B* Arg47His variant (rs1229984) in isolation. This variant is arguably the strongest and most established genetic predictor of self-reported alcohol use in European populations with a frequency of about 0.5% (Northern Europe) to 4% (Southern Europe) [24, 25]. The variant was directly genotyped using the UK Biobank array and thus no imputation of this variant was necessary. Second, we used a set of up to 25 variants including rs1229984 that had previously been associated with alcohol use and alcohol use disorder in Million Veterans Program; thus, discovery of the instruments used was entirely independent of the UK Biobank (Table A in S3 Text).

### Statistical analysis

We characterized the observational relationship between alcohol use and continuous variable risk factors using linear regression for quantitative traits, logistic regression for type 2 diabetes, and Cox proportional hazards regression for cardiovascular events or death. The reference group for all observational analyses was current non-drinkers. For our Cox analyses, we defined the start of follow up as time of enrollment into the UK Biobank study and excluded those with a cardiovascular event prior to the questionnaire to minimize survivor bias. We created two models for each outcome in our observational analysis, one model minimally adjusted for typical epidemiologic covariates (sex, age, region of recruitment as a proxy for locality, socioeconomic status, ethnicity, and smoking status; for biomarker outcomes, fasting status was also included) and one model additionally adjusted for heart disease risk factors as well as medications that affect those risk factors (BMI and waist circumference, in all models other than those for anthropometric measures; SBP and DBP, in all models other than those for blood pressure; HbA1C and diabetic medications, in models other than for type 2 diabetes; LDL, HDL, and triglycerides, in all models other than those for lipids; and lipid-lowering, or anti-hypertensive medications) (Table A in S2 Text).

We included all non-related individuals of European descent (for sample independence and to avoid population stratification) in the UK Biobank for our MR analyses. We collapsed heterozygous and homozygous carriers of rs1229984 into one group (dominant model), quantified the strength of the instrumental variable using ANOVA, and tested the relationship between the instrument variable and alcohol consumption using linear regression. We performed a one-sample MR with individual level UK Biobank data to estimate the causal effect on traits of consuming one additional drink per day on average. We used both (1) the two-stage least squares (2SLS) method with the single SNP IV and (2) the inverse variance weighted (IVW) method with the 25 SNP IV. Given possible differences in drinking patterns by sex and smoking status, we also conducted stratified analyses in male, female, current smoker, and never smoker subgroups. We used the Cochran’s Q test for heterogeneity of odds ratios to test whether outcomes varying substantially between strata were significantly different [26]. We also performed a multivariable MR (MVMR) analysis for event outcomes with any positive findings. This analysis tested the third assumption of MR (specifically, whether alcohol’s effect on these outcomes was independent of possible upstream effect or via another path) using *a priori* related factors. Lastly, we conducted an analysis of hemoglobin, hematocrit, and mean corpuscular volume levels by instrument variable status as an additional check on the potential confounding effects of anemia for our association analyses involving HbA1C.

For replication in other datasets, we collected summary statistics for the largest available study of European or mostly European ancestry for each outcome and IV (S1 Table) [27–44]. We used these summary statistics for a two-sample MR, calculating the ratio of each summary statistic coefficient to the exposure-IV coefficient from the UK Biobank. We also used the multiple SNP IV for a two-sample IVW analysis using GWAS summary statistics.

Association analyses were performed using plink 2.0, and other analyses were conducted in R 3.6.3 including the MendelianRandomization package.

## Results

UK Biobank participant characteristics are described in Tables 1 and S2. At recruitment, 92% of participants consumed alcohol. About half of participants reported drinking alcohol 1–4 times per week, and an additional one fifth of participants reported drinking daily. On average, participants reported drinking 7.7 (± 9.4) glasses per week.

**Table 1:** Summary of characteristics of UK Biobank participants by analysis dataset.

Variable	Total dataset	Observational analysis	Mendelian randomization
n	502536	481150	337484
Age (years)	67.3 (8.1)	67 (8.1)	67.6 (8)
Townsend index	-1.3 (3.1)	-1.3 (3.1)	-1.6 (2.9)
Sex			
Female	273402 (54.4)	267459 (55.6)	181236 (53.7)
Male	229134 (45.6)	213691 (44.4)	156248 (46.3)
Region			
England	445883 (88.7)	427194 (88.8)	297645 (88.2)
Wales	20808 (4.1)	19936 (4.1)	14824 (4.4)
Scotland	35845 (7.1)	34020 (7.1)	25015 (7.4)
Ethnic group			
White	472725 (94.1)	452534 (94.1)	337484 (100)
Asian or Asian British	11456 (2.3)	10847 (2.3)	0 (0)
Black or Black British	8061 (1.6)	7863 (1.6)	0 (0)
Mixed or other	7517 (1.5)	7279 (1.5)	0 (0)
Don’t know/refused	2777 (0.6)	2627 (0.5)	0 (0)
Smoking status			
Never-smoker	273537 (54.4)	265259 (55.1)	183826 (54.5)
Current smoker	52979 (10.5)	50453 (10.5)	33977 (10.1)
Former smoker	173070 (34.4)	162687 (33.8)	118505 (35.1)
No response	2950 (0.6)	2751 (0.6)	1176 (0.3)
Drinking status			
Never-drinker	22388 (4.5)	21234 (4.4)	10392 (3.1)
Current drinker	460386 (91.6)	441728 (91.8)	315257 (93.4)
Former drinker	18108 (3.6)	16631 (3.5)	11543 (3.4)
No response	1654 (0.3)	1557 (0.3)	292 (0.1)
Drinking frequency			
Daily or almost daily	101774 (20.3)	97438 (20.3)	72270 (21.4)
Three or four times a week	115445 (23)	111123 (23.1)	81462 (24.1)
Once or twice a week	129297 (25.7)	124157 (25.8)	88747 (26.3)
One to three times a month	55858 (11.1)	53724 (11.2)	37367 (11.1)
Special occasions only	58012 (11.5)	55286 (11.5)	35411 (10.5)
Never	40648 (8.1)	38009 (7.9)	21991 (6.5)
No response	1502 (0.3)	1413 (0.3)	236 (0.1)
Alcohol (weekly equivalent glasses of wine)	7.7 (9.4)	7.7 (9.4)	8.1 (9.5)
Systolic blood pressure (mmHg)	139.7 (19.7)	139.7 (19.7)	140.2 (19.7)
Diastolic blood pressure (mmHg)	82.2 (10.7)	82.3 (10.7)	82.2 (10.7)
Body mass index (kg/m^2)	27.4 (4.8)	27.4 (4.8)	27.4 (4.7)
Waist circumference (cm)	90.3 (13.5)	90 (13.4)	90.3 (13.5)
Body fat percentage	31.5 (8.5)	31.5 (8.6)	31.4 (8.5)
Cholesterol (mmol/L)	5.7 (1.1)	5.7 (1.1)	5.7 (1.1)
LDL (mmol/L)	3.6 (0.9)	3.6 (0.9)	3.6 (0.9)
HDL (mmol/L)	1.4 (0.4)	1.5 (0.4)	1.5 (0.4)
Triglycerides (mmol/L)	1.7 (1)	1.7 (1)	1.8 (1)
HbA1c (mmol/mol)	36.1 (6.8)	36 (6.5)	36 (6.5)
Glucose (mmol/L)	5.1 (1.2)	5.1 (1.2)	5.1 (1.2)
Type 2 diabetes	25217 (5)	22083 (4.6)	15493 (4.6)
Coronary heart disease	24047 (4.8)	11297 (2.3)	16102 (4.8)
All stroke	11785 (2.3)	6857 (1.4)	8044 (2.4)
Ischemic stroke	5081 (1)	3217 (0.7)	3427 (1)
Hemorrhagic stroke	2814 (0.6)	1608 (0.3)	1877 (0.6)
Heart failure	8956 (1.8)	5128 (1.1)	5921 (1.8)
Atrial fibrillation	22197 (4.4)	14292 (3)	15483 (4.6)
Any cardiovascular disease	52219 (10.4)	30833 (6.4)	35499 (10.5)
All-cause death	20284 (4)	17635 (3.7)	13700 (4.1)
ADH1B status			
Wildtype	458807 (91.3)	439084 (91.3)	322519 (95.6)
Carrier	26534 (5.3)	25593 (5.3)	14777 (4.4)
Homozygous minor allele	1979 (0.4)	1928 (0.4)	188 (0.1)

N (%) for categorical variable traits or mean (standard deviation) for quantitative variable traits (in listed units)

Exclusion of those with prior CVD (n = 21,386) yielded a dataset of 481,150 (Table 1). For our observational analysis (S1, S2 Figs, Tables A-F in S4 Text), increased alcohol use was directly related to higher systolic and diastolic blood pressure, total cholesterol, HDL, and atrial fibrillation in the fully adjusted model. We observed a J-shape association (compared with no drinking, equal or lower coefficient/odds ratio/hazard ratio with moderate drinking, but higher with heavy drinking) with BMI, waist circumference, body fat percentage, LDL, stroke (total, ischemic, and hemorrhagic), and all-cause death. Triglycerides, type 2 diabetes, myocardial infarction, heart failure and total cardiovascular disease also had a J-shape association, but with lower betas/odds ratio/hazard ratio at all drinking levels compared with non-drinkers. Alcohol was inversely associated with HbA1C.

**Fig 1:**
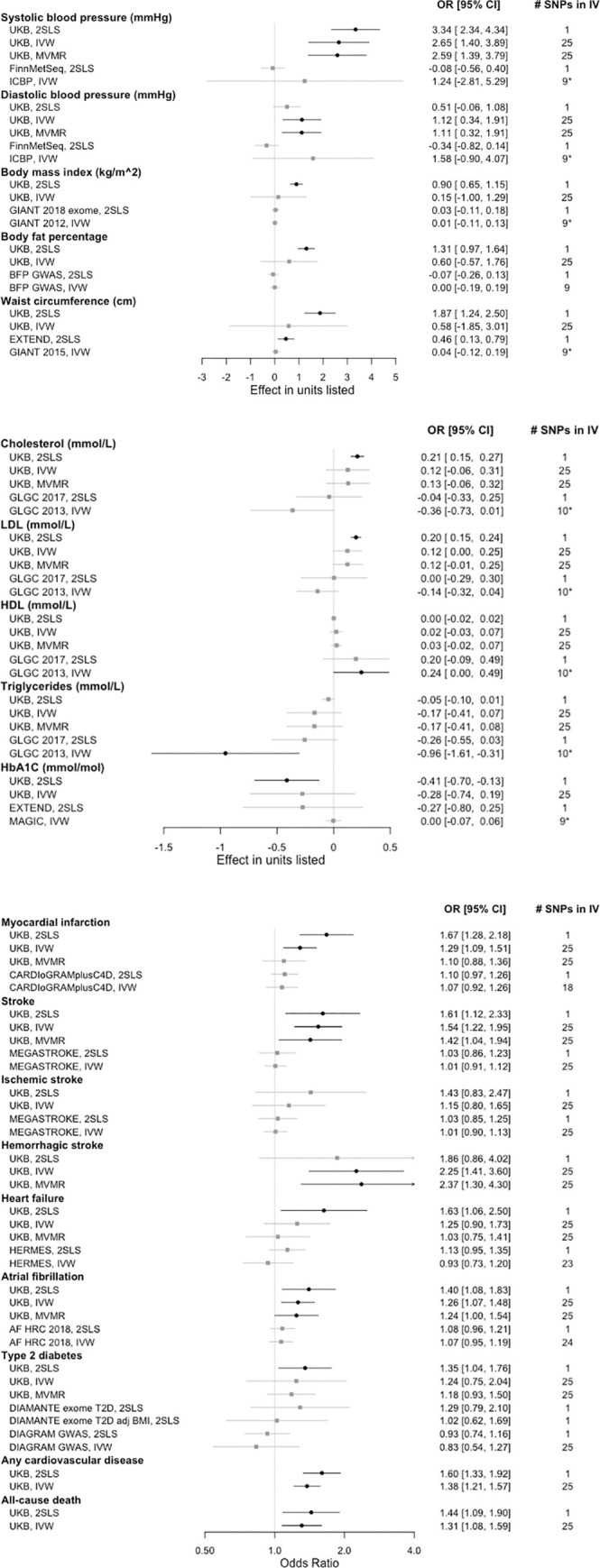
Results from Mendelian randomization (MR) of outcome variables in UK Biobank and external datasets. MR results for blood pressure and anthropometric measures (a), lipid and glycemic biomarkers (b), as well as disease outcomes and death (c). Mendelian randomization analysis outcomes for two-stage least squares (2SLS), inverse variance weighted (IVW), and multivariate Mendelian randomization (MVMR) methods in the UK Biobank (UKB) or external datasets. Beta or odds ratio (OR) [95% confidence interval (CI)] for one additional drink/day as instrumented by rs1229984 or by the indicated number of SNPs present from the 25 SNP instrumental variable (IV) set. Significant results shown in black with circles; non-significant results shown in gray with squares. * = the strongest SNP, rs1229984, was not available in the listed dataset. Sample sizes: FinnMetSeq 19,291; GIANT (2018) 449,889; GIANT (2012) 133,154; BFP GWAS 100,716; EXTEND 7,159; GIANT (2015) 232,101; GLGC (2017) 300,000; GLGC (2013) 188,577; MAGIC 123,665. Case numbers: CARDIOGRAMplusC4D 60,801; MEGASTROKE 67,162 (all stroke) and 60,341 (ischemic stroke); HERMES 47,309; AF HRC 65,446; DIAMANTE 48,286; DIAGRAM 26,676.

The Arg47His *ADH1B* variant was found in 4.4% of individuals in the UK Biobank, with only 188 subjects found to be homozygous for the variant. Our MR analysis (n = 337,484) showed that carriers of the wildtype consumed 2.1 drinks/week more than carriers of the Arg47His variant in *ADH1B* (7,127 g/year or 8.2 glasses/week for wildtype vs 5,276 g/year or 6.1 glasses/week for carriers; F = 718) (Tables 2 and S2). All 25 SNPs of the second instrumental variable investigated were found in the UK Biobank, while only 9 to 10 SNPs were present in the external datasets ICBP, both GIANT GWAS, body fat percentage GWAS, MAGIC, and GLGC. The remaining external datasets included nearly all of the IV SNPs (Figs 1A–1C, Tables A-C in S5 Text).

**Table 2:** Alcohol consumption in equivalent glasses of wine per week by group and ADH1B status.

Group	Wildtype, mean (SD) glasses/week	Variant, mean (SD) glasses/week	Increase for wildtype, glasses/week
all	8.2 (9.5)	6.1 (7.6)	2.1
male	11.2 (11.3)	8.4 (8.8)	2.8
female	5.5 (6.7)	3.9 (5.4)	1.6
current smoker	11.1 (13.4)	7.6 (9.9)	3.5
never-smoker	6.5 (7.8)	4.6 (6.1)	1.9

Mean (standard deviation) per category. p value for difference between wildtype and variant was < 2 x 10^−16^ for all rows.

Systolic blood pressure was elevated for UK Biobank analyses with both IVs, including in MVMR analyses adjusted for BMI, but this finding was not replicated in FinnMetSeq and was not statistically significant in ICBP (Fig 1A, Tables A and D in S5 Text). BMI showed an increase due to drinking per the UK Biobank 2SLS analysis but null results otherwise. Body fat percentage showed a positive association with the UK Biobank 2SLS analysis only. Waist circumference was increased with alcohol use per both UK Biobank and external 2SLS tests, but not with either IVW analysis.

Total cholesterol and LDL showed a positive association with alcohol via the UK Biobank 2SLS analysis, but these were null for further tests, including in multivariable analysis (Fig 1B, Tables B and D in S5 Text). There was a positive association between HDL and alcohol in the GLGC IVW analyses, but all HDL associations in the UK Biobank were not significant, including the MVMR analysis. Triglycerides showed a negative point estimate with alcohol for all analyses, a result that was significant only for the GLGC IVW analysis. HbA1C also had negative point estimates for the relationship with alcohol for all analyses, though all except the UK Biobank 2SLS were null.

Myocardial infarction was positively related to alcohol in the UK Biobank 2SLS and IVW analyses, but this association did not hold in the MVMR analysis in which blood pressure approached significance, nor was it replicated in CARDIOGRAMplusC4D (Fig 1C, Tables C and E in S5 Text). All stroke was positively associated with alcohol in all UK Biobank analyses including the MVMR, but null in MEGASTROKE. While ischemic stroke analyses were all null, hemorrhagic stroke was positively associated with alcohol use in UK Biobank IVW and MVMR analyses. Heart failure was positively associated with alcohol use in the initial UK Biobank analysis, but associations were null thereafter. Atrial fibrillation showed a positive association with alcohol in all UK Biobank analyses including the MVMR, but two-sample MR associations were null. Type 2 diabetes showed a positive association with alcohol in the UK Biobank 2SLS analysis, but this association disappeared in the MVMR where BMI was the significant factor. The DIAMANTE exome 2SLS result was also attenuated when adjusted for BMI. Type 2 diabetes associations in DIAGRAM were null. Alcohol use was positively associated with any cardiovascular disease and all-cause death.

Drinking varied by sex (men: 11.1 glasses/week vs. women: 5.4 glasses/week) and by smoking status (current smokers: 9.8 glasses/week vs. never-smokers: 6.5 glasses/week) in stratified analyses (Table 2, p<2e-16 for all). All strata showed an increase in systolic blood pressure with alcohol use for both MR methods. Anthropometric measurements, total cholesterol, and LDL were also elevated in all strata in the 2SLS analysis, but these results were not universally found in the IVW analysis. For the disease outcomes, women had no increased risk with alcohol use, while men had an increased risk of myocardial infarction, stroke, atrial fibrillation, any cardiovascular disease, and death in both MR analyses. However, the p-values for heterogeneity showed no difference by sex. Never smokers had higher odds ratios and more statistically significant results than current smokers for nearly all disease outcomes, but P-values for heterogeneity showed no consistent difference by smoking strata except for a borderline case for HDL. None of the disease outcomes had a protective effect from alcohol for any strata (Tables F-I in S5 Text).

Analysis of blood count data by rs1229984 carrier status showed that those with the wildtype, who drink more, had a lower hemoglobin (-0.02 g/dL, p = 0.0018) and hematocrit (-0.09%, p = 0.0024), and a higher mean corpuscular volume (+0.27 fL, p = 2.4 x 10^−13^) (Table J in S5 Text).

## Discussion

Our principal analyses within the UK Biobank suggest that alcohol use is positively associated with blood pressure, hemorrhagic stroke, atrial fibrillation, any cardiovascular disease, and all-cause death. The association between alcohol and myocardial infarction appears to be driven by other factors such as blood pressure, and the association between alcohol and type 2 diabetes appears to be driven by BMI. In summary statistics from external datasets, alcohol was predictive only of increased waist circumference (EXTEND), increased HDL (GLGC), and decreased triglycerides (GLGC), with other associations null. Associations between alcohol and anthropometric measures, total cholesterol, LDL, ischemic stroke, and heart failure were null or inconsistent between UK Biobank and external datasets. Our MR findings that alcohol is associated directly with blood pressure and atrial fibrillation were also supported by observational analyses. Our MR analyses gave discrepant results when compared to the analogous observational analysis for other phenotypes, which suggests the presence of residual confounding from unmeasured factors in observational analysis and counters the hypothesis of a protective effect of alcohol.

Two findings in our analyses are inconsistent with the hypothesis of a causal negative effect on cardiometabolic outcomes mediated through risk factors. First, we found alcohol to be causally associated with a lower level of triglycerides in GLGC, as well as a negatively trending association in the UK Biobank, which would be expected to reduce the risk of atherosclerosis related outcomes. We note this finding is contrary to what has been observed experimentally [45] but consistent with other MR studies [46–48]. Although multiple MR studies suggest triglycerides are causally associated with CVD, clinical trials of triglyceride-lowering agents have not consistently supported this relationship, leading to the conclusion that how triglycerides are lowered plays an important role in whether that lowering translates to cardiovascular benefit. Second, we found alcohol to be inversely associated with HbA1C in our observational and 2SLS MR analyses in the UK Biobank and trending towards an inverse association in the IVW analysis as well, which would be expected to reduce the risk of Type 2 diabetes. We suspect this counterintuitive set of associations related to glycemia may be a technical artifact driven by the presence of a mild (possibly nutritional) macrocytosis we observed among participants not carrying the minor allele at rs1229984 that biases HgA1C levels downwards without truly altering the risk of diabetes [49, 50].

Our stratified analyses found statistically significant MR associations among men for nearly all disease outcomes. Associations were less strong and often not significant for women but directionally consistent. Tests for heterogeneity showed no statistically significant difference between sex stratified results. Wider confidence intervals for women could simply be due to a smaller proportion of cases in women for cardiovascular diseases. Larger samples sizes are needed to more reliably document a statistically significant modification of effect of alcohol between men and women. If confirmed, these findings would suggest that causal negative effects of alcohol may only begin to express themselves at a higher consumption level despite differences in body surface area and rate of metabolism between females and males. We also observed significant results for a positive association between several disease outcomes and alcohol in never smokers, but not in current smokers. Tests for heterogeneity between smoking strata showed no statistically significant difference, and our results may simply be due to wider confidence intervals from a smaller group of current smokers.

Our findings are largely consistent with the existing literature of MR studies of alcohol and cardiovascular risk factors and outcomes which have found alcohol to be generally harmful and/or neutral for CVD related outcomes and most cardiometabolic risk factors [46–48, 51, 52]. For example, the first large MR study of risk factors and outcomes using data gathered from over 56 cohort studies of individuals of European ancestry and the same genetic instrument in *ADH1B* found moderate alcohol use to be associated with higher systolic blood pressure, waist circumference, BMI, LDL, and risk of coronary heart disease [47]. Another smaller MR study in Danes also found a direct association with a higher BMI [46]. A study of the China Kadoorie biobank (>500,000) used a combination of instruments in both *ALDH2* and *ADH1B* found alcohol to be positively associated with increasing systolic blood pressure, HDL, ischemic stroke, and intracerebral hemorrhage, but no effect was found for myocardial infarction [51]. Another recent study of alcohol and CVD outcomes in the UK Biobank using a multi-SNP instrument variable found an increase in blood pressure, stroke, and peripheral artery disease [48]. The inverse association of alcohol with triglycerides that we observed has also been shown in several other MR studies [46–48] and could be related to the HDL raising effects. Diabetes has previously been found to have no association with alcohol in an MR meta-analysis (>14,000 cases) as instrumented by the same variant we used [47]. However, another study in a Chinese population using the *ALDH2* rs671 variant, which has a more profound effect on alcohol intake, found a higher risk of diabetes with increasing alcohol [52]. Our study adds to the existing literature by showing sex-stratified differences which merit further investigation.

Major strengths of our study include the use of a single SNP as a genetic instrument to predict causality of alcohol consumption combined with a large sample size. Using a single instrument within an alcohol metabolizing gene that is strongly associated with the exposure maximizes the probability that the all assumptions of an MR study have been met and the results are accurately reflecting a relationship that is free from any residual confounding [53]. Further, we have replicated this analysis with a 25 SNP instrumental variable for alcohol use discovered in a dataset independent of the UK Biobank. A potential weakness may be the generalizability of our study given the well-established healthy cohort effect observed for the UK Biobank [54], although the healthy cohort may have helped by minimizing the inclusion of subjects with alcohol use disorder and/or moderate but still high-risk use of alcohol (e.g., binge drinkers). Our results are also limited to UK residents and therefore may vary somewhat in other populations although MR studies to date in other populations including Chinese are largely consistent with our findings [46, 47, 51, 52]. More research is needed on the determination of the causal effects of alcohol consumption in race/ethnic groups other than Europeans and East Asians to determine if effects observed to date generalize to all major race/ethnic groups. Some of our findings were not replicated in external datasets, which could reflect differences in drinking patterns between the cohorts. Two-sample analyses would be biased toward the null in the case that an external dataset has a smaller proportion of drinkers or could show a greater effect with a dataset with more drinkers.

Proposed mechanisms for the negative effect of alcohol on cardiometabolic disease include a pathway via raised blood pressure [55] and atherogenic lipids [56] as well as increased adiposity and subsequent risk of type 2 diabetes, consistent with our MR findings and those of others. Raised HDL has been proposed as a protective factor, but our MR results do not conclusively support that such elevations are directly related to alcohol among a population of predominantly moderate alcohol users. The same relationship with HDL has been observed in other MR studies [47, 48, 52]. Additionally, multiple lines of evidence now suggest that HDL levels are not causally associated with heart disease [17, 18] but instead serve as a marker of a variety of factors that may or may not affect the risk of CVD. In this context, one can speculate that physical activity raises HDL in a health-promoting way [57], while alcohol consumption does not. Further harm of alcohol for stroke could come from alcohol induced thrombocytopenia (hemorrhagic stroke) and reduced fibrinolysis (ischemic stroke) with alcohol use [58].

In conclusion, our analysis adds to the mounting evidence using MR that alcohol use does not improve cardiovascular health even in moderate amounts and likely worsens it when all other factors are considered. Given this evidence and the fact that alcohol is implicated in a number of public health concerns not directly related to cardiometabolic health, including addictive disorders, accidents, suicides, liver disease, and various types of cancers (e.g. esophageal, gastrointestinal, head and neck) [59], we believe it is time to reconsider current public health recommendations in the US and other countries which suggest that up to two drinks/day for men and one drink/day for women is not harmful and possibly beneficial to cardiovascular health [60]. This reconsideration is also supported by a more recent observational study that considered the full spectrum of alcohol related health consequences across the entire age spectrum and concluded that the level of consumption that minimizes health loss is zero [59]. Properly conducted randomized control trials [61] may one day more reliably inform us on this matter but, until that time comes, Mendelian randomization analyses provides an acceptable alternative to help inform health policy in this respect.

## Supporting information

S1 Text Disease definitions by ICD code or questionnaire variables. ICD codes used for disease definitions. ICD codes found in primary and secondary diagnoses were included. * Including all subcategories.
(PDF)

S2 Text Details of observational analysis and covariates. (PDF)

S3 Text Details of Mendelian randomization methods. Variants used for instrumental variable in inverse variance weighted, multivariable, and two-sample MR analyses; data sources for two sample Mendelian randomization.
(PDF)

S4 Text Outcomes of observational analyses. Glasses/day indicates average number of equivalent glasses of wine per day calculated from annualized intake of all alcohol types. Multiple comparisons corrections with ANOVA: * = significant for alcohol consumption via Holm method, ** = also significant with Bonferroni correction. All results are compared to a reference of no alcohol consumption. Betas and (95% confidence intervals). SBP: Systolic blood pressure (mmHg). DBP: Diastolic blood pressure (mmHg). BMI: Body mass index (kg/m^2). WAIST: Waist circumference (cm). BFP: Body fat percentage. CHOL: Cholesterol (mmol/L). LDL: Low-density lipoprotein (mmol/L). HDL: High-density lipoprotein (mmol/L). TG: Triglycerides (mmol/L). HBA1C: Glycated hemoglobin (mmol/mol). T2D: type 2 diabetes. MI: myocardial infarction. ALLSTROKE: all types of stroke. ISTROKE: ischemic stroke. HSTROKE: hemorrhagic stroke. HF: heart failure. AFIB: atrial fibrillation. CVD: any cardiovascular disease (MI, ALLSTROKE, ISTROKE, HSTROKE, HF, AFIB). Death: all-cause death.
(PDF)

S5 Text All Mendelian randomization results. (PDF)

S1 Table Data sources for two sample Mendelian randomization. * indicates the source also includes UK Biobank data. CVDKP = Cardiovascular disease knowledge portal [43], http://www.broadcvdi.org/home/portalHome. T2DKP = Type 2 diabetes knowledge portal [44], http://www.type2diabetesgenetics.org/.
(PDF)

S2 Table Descriptive statistics for variables in the stratified datasets for Mendelian randomization. n (%) for categorical variable traits or mean (standard deviation) for quantitative variable traits (in listed units). Total column is repeated from main Table 1.
(PDF)

S1 Fig Observational analysis results for effect size for given drinking category (units listed with abbreviations) for fully adjusted model. All results are compared to a reference of no alcohol consumption. Bars indicate 95% confidence interval. SBP: Systolic blood pressure (mmHg). DBP: Diastolic blood pressure (mmHg). BMI: Body mass index (kg/m^2). WAIST: Waist circumference (cm). BFP: Body fat percentage. CHOL: Cholesterol (mmol/L). LDL: Low-density lipoprotein (mmol/L). HDL: High-density lipoprotein (mmol/L). TG: Triglycerides (mmol/L). HBA1C: Glycated hemoglobin (mmol/mol).
(TIFF)

S2 Fig Observational analysis results for hazard ratio (event outcomes) or odds ratio (type 2 diabetes) for fully adjusted model. All results are compared to a reference of no alcohol consumption. Bars indicate 95% confidence interval. T2D: type 2 diabetes. MI: myocardial infarction. ALLSTROKE: all types of stroke. ISTROKE: ischemic stroke. HSTROKE: hemorrhagic stroke. HF: heart failure. AFIB: atrial fibrillation. CVD: any cardiovascular disease (MI, ALLSTROKE, ISTROKE, HSTROKE, HF, AFIB). Death: all-cause death.
(TIFF)
