# Supplementary material for: Alcohol use and cardiometabolic risk in the UK Biobank: A Mendelian randomization study
Source: PLoS One. 2021 Aug 11;16(8):e0255801. doi: 10.1371/journal.pone.0255801 (PMC8357114; doi:10.1371/journal.pone.0255801)
Supplement: S1 Text — ICD codes used for disease definitions. ICD codes found in primary and secondary diagnoses were included. * Including all subcategories. (PDF) [file pone.0255801.s001.pdf]

Table A. ICD codes used for disease definitions

Event	ICD-9	ICD-10	OPCS-4
Myocardial infarction	410*, 411*	I20.0, I21*, I22*	K40*, K41*, K42*, K43*, K44*, K45*, K46*, K49*, K50*, K75*
All stroke	430*, 431*, 432*, 433*, 434*, 435*, 436*	I60*, I61*, I62*, I63*, G45*, I64*	n/a
Ischemic stroke	433*, 434*	I63*	n/a
Hemorrhagic stroke	430*, 431*, 432*	I60*, I61*, I62*	n/a
Heart failure	428*	I50*	n/a
Atrial fibrillation	427.3*	I48*	K52.1, K62.2, K62.3, K62.4

* Including all subcategories. ICD codes found in primary and secondary diagnoses were included.

<u>Definition of diabetes</u>

Diabetes was defined using self-reported diabetes (question # 1221, 1222, 1223), self-reported medical conditions (question # 20002), self-reported medications (question # 20003), HbA1C results (question # 30750), age of diagnosis (question # 2976), started insulin within one year of diagnosis (question # 2986), diabetes diagnosed by a doctor (question # 2443), diabetes gestational only (question # 4041).

We flagged individuals who had suspected Type 1 diabetes. We considered individuals to have Type 2 diabetes if they were not flagged for Type 1, and any of the following was true:

- HbA1C exceeded threshold of 48 mmol/mol
- Type 2 diabetes was in their self-reported medical conditions
- Diabetes medications were self-reported
- Diabetes had been diagnosed by a doctor but was not gestational only

We flagged individuals for suspected type 1 diabetes if:

- Type 1 diabetes was in reported conditions, OR
- Diabetes was diagnosed before age 35, OR
- Insulin began within a year of diagnosis.
