# Supplementary material for: Alcohol use and cardiometabolic risk in the UK Biobank: A Mendelian randomization study
Source: PLoS One. 2021 Aug 11;16(8):e0255801. doi: 10.1371/journal.pone.0255801 (PMC8357114; doi:10.1371/journal.pone.0255801)
Supplement: S2 Text — (PDF) [file pone.0255801.s002.pdf]

In the partially adjusted model, we used the Townsend index to adjust for socioeconomic status. Ethnicity was grouped by the highest tree-structure group of the 21000 variable. Region was encoded as England, Scotland, or Wales. Antihypertensive and lipid-lowering medications were extracted from variables 6153 and 6177. Diabetes medications came from variables 6153, 6177, and 20003. Sex, age, and smoking status also came from survey data. Fasting time (variable 74) was also used in all biomarker outcome models.

Table A. Covariates used in observational models for given outcomes, with strikethrough indicating omitted covariates for that outcome

**Outcome**	**Fully adjusted includes all the partially adjusted plus:**
BMI, waist circumference, body fat percentage	HbA1c, diabetes medications, ~~BMI, waist circumference,~~ SBP, DBP, anti-hypertensive, LDL, HDL, triglycerides, lipid-lowering
SBP and DBP	HbA1c, diabetes medications, BMI, waist circumference, ~~SBP, DBP~~, anti-hypertensive, LDL, HDL, triglycerides, lipid-lowering
Type 2 diabetes	~~HbA1c, diabetes medications,~~ BMI, waist circumference, SBP, DBP, anti-hypertensive, LDL, HDL, triglycerides, lipid-lowering
Cholesterol, LDL, HDL, triglycerides	HbA1c, diabetes medications, BMI, waist circumference, SBP, DBP, anti-hypertensive, ~~LDL, HDL, triglycerides~~, lipid-lowering
HBA1C	~~HbA1c~~, diabetes medications, BMI, waist circumference, SBP, DBP, anti-hypertensive, LDL, HDL, triglycerides, lipid-lowering
Heart disease events (CHD, stroke, etc)	HbA1c, diabetes medications, BMI, waist circumference, SBP, DBP, anti-hypertensive, LDL, HDL, triglycerides, lipid-lowering

The differences between alcohol consumption categories in these regression models were assessed using one-way ANOVA. Given 19 outcomes evaluated with two models each, the

critical test cutoff of 0.05/38 was 0.00132. We have presented statistical significance corrected with Bonferroni and Holm adjustments for multiple comparisons.
