# Supplementary material for: Alcohol use and cardiometabolic risk in the UK Biobank: A Mendelian randomization study
Source: PLoS One. 2021 Aug 11;16(8):e0255801. doi: 10.1371/journal.pone.0255801 (PMC8357114; doi:10.1371/journal.pone.0255801)
Supplement: S3 Text — Variants used for instrumental variable in inverse variance weighted, multivariable, and two-sample MR analyses; data sources for two sample Mendelian randomization. (PDF) [file pone.0255801.s003.pdf]

All SNPs with an association with the AUDIT-C score or with Alcohol Use Disorder were assessed as potential instrument variables for alcohol use. We calculated the association between each SNP and alcohol consumption in the UK Biobank, adjusted for age at censoring (age when the data was extracted or death) and sex. Given that the phenotypes were not an exact match, we chose a generous cutoff of $p = 0.5$ for instruments to include in the analysis. Associations were similarly calculated between each SNP and outcome, age and sex adjusted. Associations between SNPs and lipids were also adjusted lipid-lowering medications, associations between SNPs and blood pressure were also adjusted for antihypertensive medications, and associations between SNPs and HbA1C were also adjusted for diabetes medications.

Table A. Variants from Million Veterans Program (MVP) evaluated for alcohol use association in UK Biobank (UKB).

rsID	chr	pos	A1 UKB	A1 freq UKB	Beta for A1 UKB	SE UKB	P UKB	L95 UKB	U95 UKB	Allele raising alcohol use MVP	Beta in UKB for the allele raising alcohol use in MVP	Included in IV
rs1260326	2	27730940	T	0.39277	-0.05430	0.00460	3.55E-32	-0.06331	-0.04529	C	0.05430	Yes
rs570436	2	45142673	C	0.44368	-0.03307	0.00481	6.44E-12	-0.04250	-0.02363	T	0.03307	Yes
rs2717071	2	58030962	G	0.37221	-0.01952	0.00455	1.76E-05	-0.02842	-0.01061	A	0.01952	Yes
rs12639940	4	39420981	G	0.37178	-0.06227	0.00456	2.24E-42	-0.07121	-0.05332	A	0.06227	Yes
rs2141284	4	99704167	A	0.00596	-0.29542	0.02102	7.43E-45	-0.33662	-0.25423	G	0.29542	Yes
rs1154431	4	100155470	G	0.04494	-0.04624	0.00779	2.88E-09	-0.06151	-0.03098	A	0.04624	Yes
rs1229984	4	100239319	T	0.02245	-0.31028	0.01077	2.32E-182	-0.33138	-0.28917	C	0.31028	Yes
rs1154433	4	100253708	G	0.41821	0.03710	0.00469	2.49E-15	0.02791	0.04628	G	0.03710	Yes
rs1229978	4	100256199	C	0.41769	0.03720	0.00468	2.03E-15	0.02802	0.04638	C	0.03720	Yes
rs78111280	4	100421822	T	0.07261	-0.02908	0.00640	5.52E-06	-0.04162	-0.01654	C	0.02908	Yes
rs57370858	4	100601316	T	0.05476	-0.02669	0.00721	2.14E-04	-0.04082	-0.01256	A	0.02669	Yes
rs140280172	4	100832564	A	0.00464	-0.25273	0.02355	7.39E-27	-0.29889	-0.20657	C	0.25273	Yes
rs150021439	4	100919254	T	0.00456	-0.25558	0.02399	1.67E-26	-0.30259	-0.20856	C	0.25558	Yes
rs186613142	4	101546509	A	0.00186	-0.22456	0.03859	5.91E-09	-0.30019	-0.14893	G	0.22456	Yes
rs13107325	4	103188709	T	0.07479	-0.05466	0.00632	5.07E-18	-0.06704	-0.04228	C	0.05466	Yes
rs62339861	4	150983421	T	0.22171	-0.00470	0.00455	0.301	-0.01361	0.00421	C	0.00470	Yes
rs2961817	5	50443063	G	0.34490	-0.00645	0.00449	0.151	-0.01526	0.00235	A	0.00645	Yes
rs185177474	5	143889333	A	0.04266	0.02527	0.00820	2.07E-03	0.00919	0.04135	A	0.02527	Yes
rs4715221	6	51359803	G	0.31079	-0.01275	0.00445	4.13E-03	-0.02146	-0.00404	A	0.01275	Yes
rs141424017	7	114951430	T	0.22675	-0.00048	0.00455	0.915	-0.00940	0.00843	T	-0.00048	No
rs17125651	10	110823083	C	0.12804	0.00347	0.00520	0.505	-0.00673	0.01367	C	0.00347	No

rs4936277	11	113431960	G	0.45463	-0.02129	0.00490	1.41E-05	-0.03091	-0.01168	A	0.02129	Yes
rs12425096	12	51980459	C	0.22154	-0.00893	0.00454	0.0494	-0.01784	-0.00002	A	0.00893	Yes
rs11075992	16	53820066	C	0.39332	-0.02516	0.00460	4.60E-08	-0.03418	-0.01614	T	0.02516	Yes
rs9937709	16	53820813	G	0.41278	-0.02497	0.00467	8.94E-08	-0.03413	-0.01582	A	0.02497	Yes
rs4794018	17	47093398	C	0.35500	0.01858	0.00451	3.75E-05	0.00975	0.02742	C	0.01858	Yes
rs35572189	17	79419025	A	0.36136	0.02247	0.00453	7.04E-07	0.01359	0.03135	A	0.02247	Yes

Betas show association with daily wine equivalents in UKB. Chr = chromosome; pos = numeric position within chromosome; A = allele; beta = effect size from regression; SE = standard deviation; p = p-value; L95 = lower limit of 95% confidence interval; U95 = upper limit of 95% confidence interval; IV = instrument variable.
