# Supplementary material for: Alcohol use and cardiometabolic risk in the UK Biobank: A Mendelian randomization study
Source: PLoS One. 2021 Aug 11;16(8):e0255801. doi: 10.1371/journal.pone.0255801 (PMC8357114; doi:10.1371/journal.pone.0255801)
Supplement: S4 Text — Glasses/day indicates average number of equivalent glasses of wine per day calculated from annualized intake of all alcohol types. Multiple comparisons corrections with ANOVA: * = significant for alcohol consumption via Holm method, ** = also significant with Bonferroni correction. All results are compared to a reference of no alcohol consumption. Betas and (95% confidence intervals). SBP: Systolic blood pressure (mmHg). DBP: Diastolic blood pressure (mmHg). BMI: Body mass index (kg/m^2). WAIST: Waist circumference (cm). BFP: Body fat percentage. CHOL: Cholesterol (mmol/L). LDL: Low-density lipoprotein (mmol/L). HDL: High-density lipoprotein (mmol/L). TG: Triglycerides (mmol/L). HBA1C: Glycated hemoglobin (mmol/mol). T2D: type 2 diabetes. MI: myocardial infarction. ALLSTROKE: all types of stroke. ISTROKE: ischemic stroke. HSTROKE: hemorrhagic stroke. HF: heart failure. AFIB: atrial fibrillation. CVD: any cardiovascular disease (MI, ALLSTROKE, ISTROKE, HSTROKE, HF, AFIB). Death: all-cause death. (PDF) [file pone.0255801.s004.pdf]

Table A. Observational analysis results (betas and 95% confidence intervals) for continuous outcomes for partially adjusted model.

Glasses/day	SBP**	DBP**	BMI**	WAIST**	BFP**	CHOL**	LDL**	HDL**	TG**	HBA1C**
>0-1	0.22 (0.07,0.36)	-0.010 (-0.09,0.07)	-1.17 (-1.21,-1.14)	-2.66 (-2.74,-2.57)	-1.11 (-1.15,-1.06)	0.11 (0.10,0.12)	0.03 (0.02,0.04)	0.11 (0.10,0.11)	-0.14 (-0.14,-0.13)	-0.99 (-1.04,-0.94)
>1-2	1.02 (0.86,1.18)	0.72 (0.62,0.81)	-1.40 (-1.44,-1.36)	-3.03 (-3.13,-2.93)	-1.31 (-1.36,-1.25)	0.19 (0.18,0.20)	0.03 (0.02,0.03)	0.20 (0.20,0.21)	-0.19 (-0.20,-0.18)	-1.60 (-1.66,-1.55)
>2-3	3.13 (2.92,3.34)	2.06 (1.94,2.18)	-1.07 (-1.12,-1.02)	-2.06 (-2.19,-1.93)	-0.79 (-0.86,-0.72)	0.28 (0.27,0.29)	0.05 (0.04,0.06)	0.26 (0.26,0.27)	-0.16 (-0.17,-0.15)	-1.82 (-1.89,-1.74)
>3	6.28 (6.05,6.51)	3.85 (3.72,3.98)	-0.69 (-0.74,-0.63)	-0.74 (-0.88,-0.59)	-0.18 (-0.25,-0.10)	0.40 (0.39,0.41)	0.07 (0.06,0.08)	0.34 (0.34,0.35)	-0.08 (-0.09,-0.06)	-1.86 (-1.94,-1.78)

Table B. Observational analysis results (betas and 95% confidence intervals) for continuous outcomes for fully adjusted model.

Glasses/day	SBP**	DBP**	BMI**	WAIST**	BFP**	CHOL**	LDL**	HDL**	TG**	HBA1C**
>0-1	0.72 (0.56,0.87)	0.48 (0.39,0.56)	-0.54 (-0.57,-0.50)	-0.97 (-1.06,-0.88)	-0.35 (-0.39,-0.30)	0.07 (0.06,0.08)	0.01 (0.00,0.02)	0.08 (0.07,0.08)	-0.07 (-0.08,-0.06)	-0.34 (-0.38,-0.29)
>1-2	1.37 (1.19,1.55)	1.21 (1.11,1.31)	-0.39 (-0.43,-0.35)	-0.32 (-0.43,-0.22)	-0.10 (-0.15,-0.04)	0.13 (0.12,0.14)	0.00 (-0.01,0.00)	0.16 (0.16,0.17)	-0.11 (-0.12,-0.10)	-0.70 (-0.75,-0.65)
>2-3	2.91 (2.67,3.14)	2.17 (2.04,2.30)	0.00 (-0.06,0.05)	0.87 (0.74,1.00)	0.49 (0.42,0.57)	0.21 (0.20,0.22)	0.01 (0.00,0.01)	0.23 (0.23,0.24)	-0.11 (-0.12,-0.10)	-0.91 (-0.98,-0.85)
>3	5.01 (4.76,5.26)	3.39 (3.24,3.53)	0.43 (0.37,0.49)	2.38 (2.23,2.52)	1.16 (1.08,1.24)	0.32 (0.30,0.33)	0.02 (0.01,0.03)	0.32 (0.32,0.32)	-0.06 (-0.08,-0.05)	-1.10 (-1.17,-1.02)

Table C. Observational analysis results (odds ratios and 95% confidence intervals) for type 2 diabetes for partially adjusted model.

**glasses/day**	**T2D****
>0-1	0.59 (0.57,0.61)
>1-2	0.44 (0.42,0.46)
>2-3	0.43 (0.4,0.45)
>3	0.47 (0.45,0.5)

Table D. Observational analysis results (odds ratios and confidence intervals) for type 2 diabetes for fully adjusted model.

**glasses/day**	**T2D****
>0-1	0.81 (0.77,0.84)
>1-2	0.69 (0.65,0.73)
>2-3	0.68 (0.63,0.73)
>3	0.75 (0.7,0.81)

Table E. Observational analysis results (hazard ratios and 95% confidence intervals) for disease events for partially adjusted model.

glasses/day	MI**	ALLSTROKE**	ISTROKE**	HSTROKE*	HF**	AFIB**	CVD**	Death**
>0-1	0.80 (0.76,0.84)	0.89 (0.84,0.95)	0.85 (0.78,0.93)	0.93 (0.82,1.05)	0.69 (0.64,0.74)	0.88 (0.84,0.92)	0.83 (0.81,0.86)	0.77 (0.74,0.80)
>1-2	0.70 (0.66,0.74)	0.83 (0.78,0.89)	0.86 (0.78,0.95)	0.79 (0.68,0.92)	0.61 (0.56,0.66)	0.89 (0.85,0.94)	0.78 (0.75,0.80)	0.71 (0.68,0.74)
>2-3	0.68 (0.63,0.72)	0.79 (0.72,0.87)	0.75 (0.65,0.86)	0.93 (0.77,1.12)	0.63 (0.57,0.70)	0.94 (0.88,0.99)	0.79 (0.75,0.82)	0.71 (0.67,0.75)
>3	0.69 (0.65,0.74)	0.98 (0.90,1.08)	1.00 (0.88,1.14)	1.07 (0.88,1.29)	0.75 (0.68,0.83)	1.10 (1.04,1.17)	0.88 (0.85,0.92)	0.91 (0.87,0.96)

Table F. Observational analysis results (hazard ratios and 95% confidence intervals) for disease events for fully adjusted model.

glasses/day	MI**	ALLSTROKE**	ISTROKE*	HSTROKE*	HF**	AFIB**	CVD**	Death**
>0-1	0.89 (0.84,0.95)	0.91 (0.85,0.98)	0.87 (0.78,0.97)	0.91 (0.78,1.06)	0.81 (0.74,0.88)	0.98 (0.93,1.03)	0.91 (0.88,0.95)	0.80 (0.76,0.84)
>1-2	0.82 (0.77,0.88)	0.87 (0.80,0.95)	0.93 (0.82,1.05)	0.76 (0.63,0.91)	0.73 (0.66,0.81)	1.00 (0.95,1.07)	0.87 (0.84,0.91)	0.77 (0.73,0.82)
>2-3	0.81 (0.74,0.88)	0.85 (0.76,0.95)	0.81 (0.69,0.95)	1.00 (0.80,1.24)	0.78 (0.68,0.88)	1.04 (0.97,1.12)	0.89 (0.85,0.93)	0.78 (0.73,0.84)
>3	0.85 (0.78,0.92)	1.02 (0.91,1.14)	1.04 (0.89,1.22)	1.00 (0.79,1.26)	0.91 (0.80,1.03)	1.20 (1.11,1.29)	0.99 (0.94,1.05)	1.04 (0.97,1.11)

Reference for all: no alcohol consumption.

Multiple comparisons corrections with ANOVA:
* = significant for alcohol consumption via Holm method, ** = also significant with Bonferroni correction. All results are compared to a reference of no alcohol consumption. Betas and (95% confidence intervals).

Abbreviations:
SBP: Systolic blood pressure (mmHg)
DBP: Diastolic blood pressure (mmHg)
BMI: Body mass index (kg/m^2)
WAIST: Waist circumference (cm)
BFP: Body fat percentage
CHOL: Cholesterol (mmol/L)
LDL: Low-density lipoprotein (mmol/L)
HDL: High-density lipoprotein (mmol/L)
TG: Triglycerides (mmol/L)
HBA1C: Glycated hemoglobin (mmol/mol)
T2D: type 2 diabetes
MI: myocardial infarction
ALLSTROKE: all types of stroke
ISTROKE: ischemic stroke
HSTROKE: hemorrhagic stroke
HF: heart failure
AFIB: atrial fibrillation
CVD: any cardiovascular disease (MI, ALLSTROKE, ISTROKE, HSTROKE, HF, AFIB)
Death: all-cause death
