# Supplementary material for: Alcohol use and cardiometabolic risk in the UK Biobank: A Mendelian randomization study
Source: PLoS One. 2021 Aug 11;16(8):e0255801. doi: 10.1371/journal.pone.0255801 (PMC8357114; doi:10.1371/journal.pone.0255801)
Supplement: S5 Text — (PDF) [file pone.0255801.s005.pdf]

Table A. Blood pressure and anthropometric outcomes

Outcome	Beta [95% CI]	# SNPs in IV
Systolic blood pressure (mmHg)		
UKB, 2SLS	3.34 [2.34, 4.34]	1
UKB, IVW	2.65 [1.40, 3.89]	25
UKB, MVMR	2.59 [1.39, 3.79]	25
FinnMetSeq, 2SLS	-0.08 [-0.56, 0.40]	1
ICBP, IVW	1.24 [-2.81, 5.29]	9*
Diastolic blood pressure (mmHg)		
UKB, 2SLS	0.51 [-0.06, 1.08]	1
UKB, IVW	1.12 [0.34, 1.91]	25
UKB, MVMR	1.11 [0.32, 1.91]	25
FinnMetSeq, 2SLS	-0.34 [-0.82, 0.14]	1
ICBP, IVW	1.58 [-0.90, 4.07]	9*
Body mass index (kg/m^2)		
UKB, 2SLS	0.90 [0.65, 1.15]	1
UKB, IVW	0.15 [-1.00, 1.29]	25
GIANT 2018 exome, 2SLS	0.03 [-0.11, 0.18]	1
GIANT 2012, IVW	0.01 [-0.11, 0.13]	9*
Body fat percentage		
UKB, 2SLS	1.31 [0.97, 1.64]	1
UKB, IVW	0.60 [-0.57, 1.76]	25
BFP GWAS, 2SLS	-0.07 [-0.26, 0.13]	1
BFP GWAS, IVW	0.00 [-0.19, 0.19]	9*
Waist circumference (cm)		
UKB, 2SLS	1.87 [1.24, 2.50]	1
UKB, IVW	0.58 [-1.85, 3.01]	25
EXTEND, 2SLS	0.46 [0.13, 0.79]	1
GIANT 2015, IVW	0.04 [-0.12, 0.19]	9*

Beta and 95% confidence interval (CI) for one additional drink per day of alcohol, and number of SNPs in instrumental variable (IV). * indicates the main SNP, rs1229984, was not available in this dataset.

Table B. Serum biomarkers

Outcome	Beta [95% CI]	# SNPs in IV
Cholesterol (mmol/L)		
UKB, 2SLS	0.21 [0.15, 0.27]	1
UKB, IVW	0.12 [-0.06, 0.31]	25
UKB, MVMR	0.13 [-0.06, 0.32]	25
GLGC 2017, 2SLS	-0.04 [-0.33, 0.25]	1
GLGC 2013, IVW	-0.36 [-0.73, 0.01]	10*
LDL (mmol/L)		
UKB, 2SLS	0.20 [0.15, 0.24]	1
UKB, IVW	0.12 [0.00, 0.25]	25

UKB, MVMR	0.12 [-0.01, 0.25]	25
GLGC 2017, 2SLS	0.00 [-0.29, 0.30]	1
GLGC 2013, IVW	-0.14 [-0.32, 0.04]	10*
HDL (mmol/L)		
UKB, 2SLS	0.00 [-0.02, 0.02]	1
UKB, IVW	0.02 [-0.03, 0.07]	25
UKB, MVMR	0.03 [-0.02, 0.07]	25
GLGC 2017, 2SLS	0.20 [-0.09, 0.49]	1
GLGC 2013, IVW	0.24 [0.00, 0.49]	10*
Triglycerides (mmol/L)		
UKB, 2SLS	-0.05 [-0.10, 0.01]	1
UKB, IVW	-0.17 [-0.41, 0.07]	25
UKB, MVMR	-0.17 [-0.41, 0.08]	25
GLGC 2017, 2SLS	-0.26 [-0.55, 0.03]	1
GLGC 2013, IVW	-0.96 [-1.61, -0.31]	10*
HbA1C (mmol/mol)		
UKB, 2SLS	-0.41 [-0.70, -0.13]	1
UKB, IVW	-0.28 [-0.74, 0.19]	25
EXTEND, 2SLS	-0.27 [-0.80, 0.25]	1
MAGIC, IVW	0.00 [-0.07, 0.06]	9*

Beta and 95% confidence interval (CI) for one additional drink per day of alcohol, and number of SNPs in instrumental variable (IV). * indicates the main SNP, rs1229984, was not available in this dataset.

Table C. Binary variable outcomes

Outcome	OR [95% CI]	# SNPs in IV
Myocardial infarction		
UKB, 2SLS	1.67 [1.28, 2.18]	1
UKB, IVW	1.29 [1.09, 1.51]	25
UKB, MVMR	1.10 [0.88, 1.36]	25
CARDIoGRAMplusC4D, 2SLS	1.10 [0.97, 1.26]	1
CARDIoGRAMplusC4D, IVW	1.07 [0.92, 1.26]	18
Stroke		
UKB, 2SLS	1.61 [1.12, 2.33]	1
UKB, IVW	1.54 [1.22, 1.95]	25
UKB, MVMR	1.42 [1.04, 1.94]	25
MEGASTROKE, 2SLS	1.03 [0.86, 1.23]	1
MEGASTROKE, IVW	1.01 [0.91, 1.12]	25
Ischemic stroke		
UKB, 2SLS	1.43 [0.83, 2.47]	1
UKB, IVW	1.15 [0.80, 1.65]	25

MEGASTROKE, 2SLS	1.03 [0.85, 1.25]	1
MEGASTROKE, IVW	1.01 [0.90, 1.13]	25
Hemorrhagic stroke		
UKB, 2SLS	1.86 [0.86, 4.02]	1
UKB, IVW	2.25 [1.41, 3.60]	25
UKB, MVMR	2.37 [1.30, 4.30]	25
Heart failure		
UKB, 2SLS	1.63 [1.06, 2.50]	1
UKB, IVW	1.25 [0.90, 1.73]	25
UKB, MVMR	1.03 [0.75, 1.41]	25
HERMES, 2SLS	1.13 [0.95, 1.35]	1
HERMES, IVW	0.93 [0.73, 1.20]	23
Atrial fibrillation		
UKB, 2SLS	1.40 [1.08, 1.83]	1
UKB, IVW	1.26 [1.07, 1.48]	25
UKB, MVMR	1.24 [1.00, 1.54]	25
AF HRC 2018, 2SLS	1.08 [0.96, 1.21]	1
AF HRC 2018, IVW	1.07 [0.95, 1.19]	24
Type 2 diabetes		
UKB, 2SLS	1.35 [1.04, 1.76]	1
UKB, IVW	1.24 [0.75, 2.04]	25
UKB, MVMR	1.18 [0.93, 1.50]	25
DIAMANTE exome T2D, 2SLS	1.29 [0.79, 2.10]	1
DIAMANTE exome T2D adj BMI, 2SLS	1.02 [0.62, 1.69]	1
DIAGRAM GWAS, 2SLS	0.93 [0.74, 1.16]	1
DIAGRAM GWAS, IVW	0.83 [0.54, 1.27]	25
Any cardiovascular disease		
UKB, 2SLS	1.60 [1.33, 1.92]	1
UKB, IVW	1.38 [1.21, 1.57]	25
All-cause death		
UKB, 2SLS	1.44 [1.09, 1.90]	1
UKB, IVW	1.31 [1.08, 1.59]	25

Odds ratio (OR) and 95% confidence interval (CI) for one additional drink per day of alcohol, and number of SNPs in instrumental variable (IV). * indicates the main SNP, rs1229984, was not available in this dataset.

Table D. Risk factor outcome MVMR results

Outcome	Beta	LCL	UCL
Cholesterol (mmol/L)			
Alcohol (per +1 drink/day)	0.128	-0.063	0.320
BMI (kg/m^2)	-0.025	-0.091	0.042
LDL (mmol/L)			
Alcohol (per +1 drink/day)	0.123	-0.005	0.251
BMI (kg/m^2)	-0.005	-0.050	0.040
HDL (mmol/L)			
Alcohol (per +1 drink/day)	0.026	-0.019	0.071
BMI (kg/m^2)	-0.023	-0.039	-0.007
Triglycerides (mmol/L)			
Alcohol (per +1 drink/day)	-0.170	-0.415	0.075
BMI (kg/m^2)	0.006	-0.079	0.092
Systolic blood pressure (mmHg)			
Alcohol (per +1 drink/day)	2.590	1.391	3.788
BMI (kg/m^2)	0.373	-0.047	0.792
Diastolic blood pressure (mmHg)			
Alcohol (per +1 drink/day)	1.115	0.315	1.915
BMI (kg/m^2)	0.062	-0.218	0.342

Beta, 95% lower confidence limit (LCL), and 95% upper confidence limit (UCL) for outcomes with positive findings in the 2SLS or inverse variance weighted Mendelian randomization analyses.

Table E. Disease outcome MVMR results

Outcome	OR	LCL	UCL
Myocardial infarction			
Alcohol (per +1 drink/day)	1.10	0.88	1.36
Systolic blood pressure (mmHg)	1.04	0.98	1.11
Smoking (ever/never)	1.35	0.77	2.37
LDL (mmol/L)	1.28	0.69	2.36
All Stroke			
Alcohol (per +1 drink/day)	1.42	1.04	1.94
Systolic blood pressure (mmHg)	1.04	0.95	1.13
Smoking (ever/never)	0.64	0.28	1.45
LDL (mmol/L)	1.10	0.45	2.68
Hemorrhagic Stroke			
Alcohol (per +1 drink/day)	2.37	1.30	4.30
Systolic blood pressure (mmHg)	0.98	0.85	1.13
Atrial fibrillation			
Alcohol (per +1 drink/day)	1.24	1.00	1.54
Systolic blood pressure (mmHg)	1.00	0.95	1.06
BMI (kg/m^2)	1.05	0.99	1.11
Type 2 diabetes			
Alcohol (per +1 drink/day)	1.18	0.93	1.50
BMI (kg/m^2)	1.46	1.35	1.59

Odds ratio (OR), 95% lower confidence limit (LCL), and 95% upper confidence limit (UCL) for outcomes with positive findings in the 2SLS or inverse variance weighted Mendelian randomization analyses.

Table F. IVW MR stratified analysis for continuous variables

Outcome	Male	Female	p value sex difference	Never smokers	Current smokers	p value smoking difference
Systolic blood pressure (mmHg)	2.62 [1.66, 3.58]	2.84 [0.61, 5.06]	0.86	2.23 [0.93, 3.52]	2.71 [1.20, 4.23]	0.63
Diastolic blood pressure (mmHg)	1.23 [0.63, 1.83]	0.91 [-0.44, 2.26]	0.67	1.37 [0.45, 2.30]	1.59 [0.69, 2.49]	0.74
Body mass index (kg/m^2)	0.18 [-0.69, 1.05]	0.26 [-1.44, 1.97]	0.93	0.28 [-0.88, 1.44]	0.15 [-0.86, 1.16]	0.87
Body fat percentage	0.56 [-0.35, 1.48]	0.79 [-0.93, 2.51]	0.82	0.67 [-0.55, 1.89]	0.68 [-0.30, 1.65]	0.99
Waist circumference	0.60 [-1.37, 2.58]	0.82 [-2.62, 4.26]	0.92	0.64 [-1.82, 3.11]	0.69 [-1.39, 2.76]	0.98
Cholesterol (mmol/L)	0.17 [0.01, 0.32]	0.04 [-0.24, 0.31]	0.42	0.08 [-0.14, 0.29]	0.07 [-0.05, 0.20]	0.99
LDL (mmol/L)	0.14 [0.04, 0.24]	0.07 [-0.12, 0.26]	0.51	0.09 [-0.05, 0.23]	0.06 [-0.02, 0.13]	0.70
HDL (mmol/L)	0.03 [-0.01, 0.06]	0.01 [-0.07, 0.10]	0.74	0.01 [-0.05, 0.06]	0.07 [0.04, 0.10]	0.06
Triglycerides (mmol/L)	-0.13 [-0.33, 0.08]	-0.23 [-0.55, 0.08]	0.59	-0.14 [-0.40, 0.12]	-0.24 [-0.41, -0.07]	0.54
HbA1C (mmol/mol)	-0.11 [-0.60, 0.37]	-0.52 [-1.00, -0.05]	0.24	-0.40 [-0.98, 0.18]	-0.45 [-0.86, -0.05]	0.89

Betas and 95% confidence intervals for strata and p-values for Cochran's Q test for heterogeneity between strata pairs (male/female and never smokers/current smokers)

Table G. 2SLS MR stratified analysis for continuous variables

Outcome	Male	Female	p value sex difference	Never smokers	Current smokers	p value smoking difference
Systolic blood pressure (mmHg)	2.95 [1.86, 4.05]	4.10 [2.22, 5.97]	0.30	2.73 [1.19, 4.28]	3.01 [1.03, 4.98]	0.83
Diastolic blood pressure (mmHg)	0.68 [0.03, 1.33]	0.29 [-0.75, 1.34]	0.54	0.45 [-0.44, 1.33]	1.54 [0.38, 2.70]	0.14
Body mass index (kg/m^2)	0.80 [0.54, 1.05]	1.05 [0.54, 1.55]	0.38	0.87 [0.48, 1.26]	0.73 [0.22, 1.23]	0.66
Body fat percentage	1.26 [0.92, 1.61]	1.37 [0.70, 2.04]	0.78	1.27 [0.74, 1.80]	1.22 [0.53, 1.90]	0.91
Waist circumference	1.75 [1.08, 2.43]	2.04 [0.83, 3.26]	0.68	1.68 [0.71, 2.64]	1.60 [0.32, 2.88]	0.92
Cholesterol (mmol/L)	0.24 [0.18, 0.30]	0.15 [0.05, 0.26]	0.16	0.15 [0.06, 0.23]	0.18 [0.06, 0.29]	0.67
LDL (mmol/L)	0.22 [0.17, 0.26]	0.16 [0.08, 0.24]	0.22	0.15 [0.08, 0.22]	0.13 [0.04, 0.22]	0.72
HDL (mmol/L)	0.00 [-0.02, 0.02]	-0.01 [-0.05, 0.03]	0.64	-0.02 [-0.05, 0.01]	0.06 [0.02, 0.10]	0.0036
Triglycerides (mmol/L)	-0.03 [-0.10, 0.04]	-0.07 [-0.16, 0.01]	0.47	0.00 [-0.08, 0.08]	-0.11 [-0.24, 0.01]	0.15
HbA1C (mmol/mol)	-0.23 [-0.59, 0.13]	-0.70 [-1.16, -0.23]	0.12	-0.61 [-1.02, -0.21]	-0.60 [-1.25, 0.05]	0.98

Betas and 95% confidence intervals for strata and p-values for Cochran’s Q test for heterogeneity between strata pairs (male/female and never smokers/current smokers)

Table H. IVW MR stratified analysis for binary variables

Outcome	Male	Female	p value sex difference	Never smokers	Current smokers	p value smoking difference
Myocardial infarction	1.22 [1.02, 1.47]	1.17 [0.75, 1.84]	0.86	1.48 [1.10, 2.01]	1.22 [0.94, 1.59]	0.35
Stroke	1.44 [1.16, 1.79]	1.63 [0.98, 2.69]	0.67	1.92 [1.30, 2.83]	1.25 [0.87, 1.78]	0.11
Ischemic stroke	1.18 [0.86, 1.63]	0.96 [0.44, 2.08]	0.62	1.30 [0.72, 2.36]	0.82 [0.51, 1.33]	0.24
Hemorrhagic stroke	1.96 [1.21, 3.18]	2.66 [0.98, 7.22]	0.59	5.35 [2.24, 12.78]	1.04 [0.49, 2.18]	0.01
Heart failure	1.28 [0.94, 1.73]	0.89 [0.47, 1.71]	0.33	1.85 [1.09, 3.15]	1.36 [0.88, 2.09]	0.38
Atrial fibrillation	1.23 [1.06, 1.43]	1.20 [0.81, 1.78]	0.90	1.55 [1.16, 2.06]	1.17 [0.84, 1.62]	0.21
Type 2 diabetes	1.23 [0.80, 1.88]	1.15 [0.60, 2.21]	0.88	1.38 [0.79, 2.39]	1.27 [0.88, 1.82]	0.80
Any cardiovascular disease	1.34 [1.18, 1.53]	1.23 [0.93, 1.63]	0.58	1.61 [1.32, 1.96]	1.29 [1.06, 1.58]	0.13
All-cause death	1.38 [1.15, 1.67]	0.99 [0.68, 1.46]	0.13	0.95 [0.67, 1.35]	1.30 [1.02, 1.67]	0.15

Odds ratios and 95% confidence intervals for strata and p-values for Cochran's Q test for heterogeneity between strata pairs (male/female and never smokers/current smokers)

Table I. 2SLS MR stratified analysis for binary variables

Outcome	Male	Female	p value sex difference	Never smokers	Current smokers	p value smoking difference
Myocardial infarction	1.67 [1.31, 2.12]	1.13 [0.57, 2.24]	0.29	1.88 [1.15, 3.08]	1.15 [0.75, 1.78]	0.15
Stroke	1.55 [1.07, 2.25]	1.59 [0.73, 3.44]	0.96	1.59 [0.84, 2.98]	1.20 [0.67, 2.17]	0.53
Ischemic stroke	1.54 [0.89, 2.65]	1.02 [0.31, 3.35]	0.54	1.46 [0.56, 3.86]	0.77 [0.35, 1.66]	0.31
Hemorrhagic stroke	1.64 [0.73, 3.70]	2.25 [0.48, 10.42]	0.72	2.42 [0.61, 9.57]	1.47 [0.41, 5.23]	0.60
Heart failure	1.86 [1.23, 2.80]	0.82 [0.31, 2.15]	0.13	1.58 [0.71, 3.52]	1.39 [0.70, 2.77]	0.81
Atrial fibrillation	1.44 [1.12, 1.85]	1.12 [0.61, 2.04]	0.45	1.35 [0.86, 2.12]	1.33 [0.78, 2.27]	0.96
Type 2 diabetes	1.36 [1.05, 1.76]	1.20 [0.68, 2.14]	0.70	1.58 [0.99, 2.52]	1.07 [0.66, 1.74]	0.26
Any cardiovascular disease	1.68 [1.40, 2.00]	1.14 [0.76, 1.72]	0.09	1.56 [1.14, 2.15]	1.37 [0.99, 1.91]	0.57
All-cause death	1.66 [1.24, 2.21]	0.92 [0.52, 1.64]	0.07	0.85 [0.52, 1.39]	1.11 [0.75, 1.65]	0.40

Odds ratios and 95% confidence intervals for strata and p-values for Cochran's Q test for heterogeneity between strata pairs (male/female and never smokers/current smokers)

Table J. Additional analysis of blood count data by instrument variable group.

Variable	Heterozygous + homozygous group	Wildtype	p-value	F statistic
Hemoglobin (g/dL)	14.23	14.21	0.0179	5.6
Hematocrit (%)	41.24	41.15	0.00237	9.2
Mean corpuscular volume (fL)	91.08	91.35	2.37e-13	53.7

Mean for each value by instrument variable status; p-value and F statistic from ANOVA.
