# Supplementary material for: Alcohol use and cardiometabolic risk in the UK Biobank: A Mendelian randomization study
Source: PLoS One. 2021 Aug 11;16(8):e0255801. doi: 10.1371/journal.pone.0255801 (PMC8357114; doi:10.1371/journal.pone.0255801)
Supplement: S1 Table — * indicates the source also includes UK Biobank data. CVDKP = Cardiovascular disease knowledge portal [43], http://www.broadcvdi.org/home/portalHome. T2DKP = Type 2 diabetes knowledge portal [44], http://www.type2diabetesgenetics.org/. (PDF) [file pone.0255801.s006.pdf]

**Name**	**Extracted summary statistics for phenotypes**	**Population ancestry**	**n (cases, where applicable)**	**From**
CARDIoGRAMplusC4D with 1000 genomes GWAS meta-analysis [27]	Myocardial infarction	Mixed	184,305 (60,801)	http://www.cardiogramplusc4d.org/data-downloads/
MEGASTROKE [28]	All stroke, Ischemic stroke	European	446,696 (67,162); 440,328 (60,341)	https://www.ebi.ac.uk/gwas/studies/GCST006906, https://www.ebi.ac.uk/gwas/studies/GCST006908
HERMES Heart Failure GWAS* [29]	Heart failure	European	972,032 (47,309)	CVDKP
2018 AF HRC GWAS [30]	Atrial fibrillation	Mixed	588,190 (65,446)	CVDKP
DIAMANTE T2D exome chip meta-analysis* [31]	Type 2 diabetes	European	298,957 (48,286)	http://diagram-consortium.org/downloads.html
DIAGRAM 1000G GWAS [32]	Type 2 diabetes	European	159,208 (26,676)	http://diagram-consortium.org/downloads.html
FinnMetSeq exome sequence analysis [33]	Systolic blood pressure, diastolic blood pressure, body fat percentage	European	19,291	T2DKP
MAGIC GWAS [34]	HbA1C	European	123,665	http://magicinvestigators.org/downloads/
GIANT 2018 BMI, Height exome chip analysis [35]	BMI	European	449,889	https://portals.broadinstitute.org/collaboration/giant/index.php/GIANT_consortium_data_files

**Name**	**Extracted summary statistics for phenotypes**	**Population ancestry**	**n (cases, where applicable)**	**From**
GIANT 2012 GWAS [36]	BMI	European	133,154	https://portals.broadinstitute.org/collaboration/giant/index.php/GIANT_consortium_data_files
GIANT 2015 GWAS [37]	Waist circumference	European	232,101	https://portals.broadinstitute.org/collaboration/giant/index.php/GIANT_consortium_data_files
International Consortium on Blood Pressure (ICBP) [38]	Systolic and diastolic blood pressure	European	201,529	https://www.ncbi.nlm.nih.gov/projects/gap/cgi-bin/analysis.cgi?study_id=phs000585.v2.p1
EXTEND GWAS [39]	Waist circumference	European	7,159	T2DKP
Body fat percentage GWAS [40]	Body fat percentage	Mixed (89,267 European)	100,716	https://www.ebi.ac.uk/gwas/studies/GCST003435
GLGC exome chip analysis [41]	Total cholesterol, LDL, HDL, triglycerides	Mixed (273,050 European)	~300,000	http://csg.sph.umich.edu/willer/public/lipids2017/
GLGC 2013 GWAS [42]	Total cholesterol, LDL, HDL, triglycerides	European	188,577	https://www.ebi.ac.uk/gwas/publications/24097068
