# Supplementary material for: Alcohol use and cardiometabolic risk in the UK Biobank: A Mendelian randomization study
Source: PLoS One. 2021 Aug 11;16(8):e0255801. doi: 10.1371/journal.pone.0255801 (PMC8357114; doi:10.1371/journal.pone.0255801)
Supplement: S2 Table — n (%) for categorical variable traits or mean (standard deviation) for quantitative variable traits (in listed units). Total column is repeated from main Table 1. (PDF) [file pone.0255801.s007.pdf]

Variable	MR - total	MR - male	MR - female	MR - nonsmokers	MR - smokers
n	337484	156248	181236	183826	33977
Sex					
Female	181236 (53.7)	0 (0)	181236 (100)	107418 (58.4)	15577 (45.8)
Male	156248 (46.3)	156248 (100)	0 (0)	76408 (41.6)	18400 (54.2)
Age (years)	67.6 (8)	67.9 (8.1)	67.4 (7.9)	66.9 (8.1)	65.9 (8.1)
Region					
England	297645 (88.2)	138232 (88.5)	159413 (88)	161683 (88)	29350 (86.4)
Wales	14824 (4.4)	6822 (4.4)	8002 (4.4)	8236 (4.5)	1614 (4.8)
Scotland	25015 (7.4)	11194 (7.2)	13821 (7.6)	13907 (7.6)	3013 (8.9)
Townsend index	-1.6 (2.9)	-1.5 (3)	-1.6 (2.9)	-1.9 (2.7)	-0.1 (3.5)
Ethnic group					
White	337484 (100)	156248 (100)	181236 (100)	183826 (100)	33977 (100)
Asian or Asian British	0 (0)	0 (0)	0 (0)	0 (0)	0 (0)
Black or Black British	0 (0)	0 (0)	0 (0)	0 (0)	0 (0)
Mixed or other	0 (0)	0 (0)	0 (0)	0 (0)	0 (0)
Don't know/refused	0 (0)	0 (0)	0 (0)	0 (0)	0 (0)
Smoking status					
Never-smoker	183826 (54.5)	76408 (48.9)	107418 (59.3)	183826 (100)	0 (0)
Current smoker	33977 (10.1)	18400 (11.8)	15577 (8.6)	0 (0)	33977 (100)
Former smoker	118505 (35.1)	60883 (39)	57622 (31.8)	0 (0)	0 (0)
No response	1176 (0.3)	557 (0.4)	619 (0.3)	0 (0)	0 (0)
Drinking status					

Never-drinker	10392 (3.1)	2623 (1.7)	7769 (4.3)	8234 (4.5)	674 (2)
Current drinker	315257 (93.4)	148479 (95)	166778 (92)	170381 (92.7)	31430 (92.5)
Former drinker	11543 (3.4)	5017 (3.2)	6526 (3.6)	5084 (2.8)	1806 (5.3)
No response	292 (0.1)	129 (0.1)	163 (0.1)	127 (0.1)	67 (0.2)
Drinking frequency					
Daily or almost daily	72270 (21.4)	41339 (26.5)	30931 (17.1)	30020 (16.3)	9136 (26.9)
Three or four times a week	81462 (24.1)	42266 (27.1)	39196 (21.6)	44049 (24)	6542 (19.3)
Once or twice a week	88747 (26.3)	40983 (26.2)	47764 (26.4)	52425 (28.5)	7865 (23.1)
One to three times a month	37367 (11.1)	13662 (8.7)	23705 (13.1)	22816 (12.4)	3720 (10.9)
Special occasions only	35411 (10.5)	10229 (6.5)	25182 (13.9)	21071 (11.5)	4167 (12.3)
Never	21991 (6.5)	7656 (4.9)	14335 (7.9)	13354 (7.3)	2491 (7.3)
No response	236 (0.1)	113 (0.1)	123 (0.1)	91 (0)	56 (0.2)
Alcohol (weekly equivalent glasses of wine)	8.1 (9.5)	11.1 (11.2)	5.4 (6.6)	6.5 (7.8)	10.9 (13.3)
Systolic blood pressure (mmHg)	140.2 (19.7)	143.2 (18.5)	137.6 (20.3)	139.7 (19.5)	137.5 (19.5)
Diastolic blood pressure (mmHg)	82.2 (10.7)	84.1 (10.5)	80.6 (10.5)	82.2 (10.6)	81.6 (10.9)
Body mass index (kg/m^2)	27.4 (4.7)	27.8 (4.2)	27 (5.1)	27.1 (4.7)	27.1 (4.8)
Waist circumference (cm)	90.3 (13.5)	97 (11.3)	84.6 (12.5)	88.7 (13.2)	91.2 (13.5)
Body fat percentage	31.4 (8.5)	25.3 (5.8)	36.6 (6.9)	31.4 (8.6)	29.9 (8.6)
Cholesterol (mmol/L)	5.7 (1.1)	5.5 (1.1)	5.9 (1.1)	5.7 (1.1)	5.7 (1.2)
LDL (mmol/L)	3.6 (0.9)	3.5 (0.9)	3.6 (0.9)	3.6 (0.9)	3.6 (0.9)
HDL (mmol/L)	1.5 (0.4)	1.3 (0.3)	1.6 (0.4)	1.5 (0.4)	1.4 (0.4)
Triglycerides (mmol/L)	1.8 (1)	2 (1.1)	1.6 (0.9)	1.7 (1)	2 (1.2)

HbA1c (mmol/mol)	36 (6.5)	36.3 (7.3)	35.7 (5.7)	35.5 (6)	37.1 (7.2)
Glucose (mmol/L)	5.1 (1.2)	5.2 (1.4)	5.1 (1)	5.1 (1.1)	5.1 (1.2)
Type 2 diabetes	15493 (4.6)	9879 (6.3)	5614 (3.1)	6402 (3.5)	1715 (5)
Coronary heart disease	16102 (4.8)	12260 (7.8)	3842 (2.1)	6057 (3.3)	2274 (6.7)
All stroke	8044 (2.4)	4825 (3.1)	3219 (1.8)	3498 (1.9)	1193 (3.5)
Ischemic stroke	3427 (1)	2195 (1.4)	1232 (0.7)	1434 (0.8)	561 (1.7)
Hemorrhagic stroke	1877 (0.6)	1002 (0.6)	875 (0.5)	811 (0.4)	278 (0.8)
Heart failure	5921 (1.8)	4116 (2.6)	1805 (1)	2145 (1.2)	920 (2.7)
Atrial fibrillation	15483 (4.6)	10425 (6.7)	5058 (2.8)	6766 (3.7)	1530 (4.5)
Any cardiovascular disease	35499 (10.5)	24023 (15.4)	11476 (6.3)	14911 (8.1)	4532 (13.3)
All-cause death	13700 (4.1)	8410 (5.4)	5290 (2.9)	5102 (2.8)	2696 (7.9)
ADH1B status					
Wildtype	322519 (95.6)	149158 (95.5)	173361 (95.7)	175669 (95.6)	32518 (95.7)
Carrier	14777 (4.4)	7013 (4.5)	7764 (4.3)	8056 (4.4)	1441 (4.2)
Homozygous minor allele	188 (0.1)	77 (0)	111 (0.1)	101 (0.1)	18 (0.1)
