# Supplementary figures and images for: Alcohol use and cardiometabolic risk in the UK Biobank: A Mendelian randomization study
Source: PLoS One. 2021 Aug 11;16(8):e0255801. doi: 10.1371/journal.pone.0255801 (PMC8357114; doi:10.1371/journal.pone.0255801)

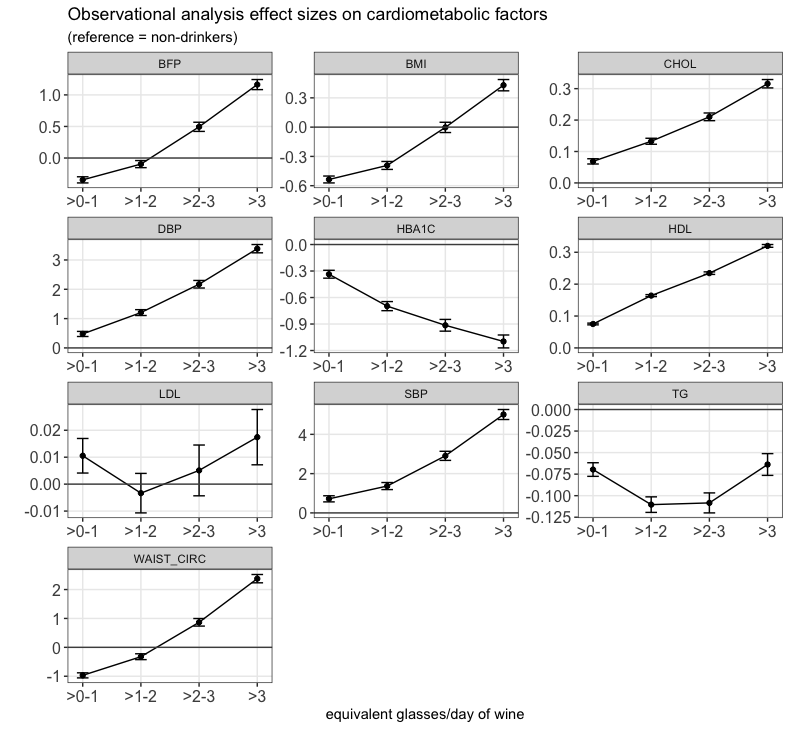

Supplement: S1 Fig — All results are compared to a reference of no alcohol consumption. Bars indicate 95% confidence interval. SBP: Systolic blood pressure (mmHg). DBP: Diastolic blood pressure (mmHg). BMI: Body mass index (kg/m^2). WAIST: Waist circumference (cm). BFP: Body fat percentage. CHOL: Cholesterol (mmol/L). LDL: Low-density lipoprotein (mmol/L). HDL: High-density lipoprotein (mmol/L). TG: Triglycerides (mmol/L). HBA1C: Glycated hemoglobin (mmol/mol). (TIFF) [file pone.0255801.s008.tiff]

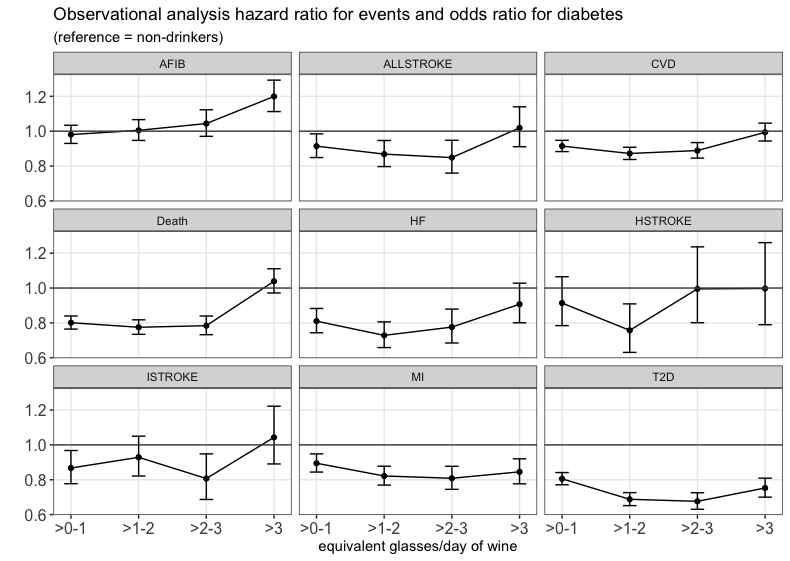

Supplement: S2 Fig — All results are compared to a reference of no alcohol consumption. Bars indicate 95% confidence interval. T2D: type 2 diabetes. MI: myocardial infarction. ALLSTROKE: all types of stroke. ISTROKE: ischemic stroke. HSTROKE: hemorrhagic stroke. HF: heart failure. AFIB: atrial fibrillation. CVD: any cardiovascular disease (MI, ALLSTROKE, ISTROKE, HSTROKE, HF, AFIB). Death: all-cause death. (TIFF) [file pone.0255801.s009.tiff]
